# Herpesvirus-Associated Visual Impairment: Clinical Features, Etiological Spectrum, and Treatment Outcomes in Consecutive Patients from a Tertiary Neurological Clinic

**DOI:** 10.3390/brainsci16070768

**Published:** 2026-07-22

**Authors:** Lei Liu, Jingxiao Zhang, Qiuying Ma, Jiawei Wang

**Affiliations:** Department of Neurology, Beijing Tongren Hospital, Capital Medical University, Beijing 100730, China; pathologyliu@mail.ccmu.edu.cn (L.L.); jingxiaobb@163.com (J.Z.); maqiuyinglf@163.com (Q.M.)

**Keywords:** herpesvirus, visual impairment, optic neuritis, acute retinal necrosis, metagenomic next-generation sequencing, pseudorabies virus

## Abstract

[Background] Herpesvirus infections can induce diverse visual impairments with permanent sequelae, yet systematic data on their clinical spectrum and outcomes remain scarce. [Methods] We conducted a single-center retrospective cohort study at the Department of Neurology, Beijing Tongren Hospital, Capital Medical University. Thirteen consecutive patients (19 affected eyes) with herpesvirus-related visual impairment admitted between January 2016 and January 2025 were enrolled. Demographic data, clinical manifestations, etiological tests (polymerase chain reaction [PCR], metagenomic next-generation sequencing [mNGS], serology), neuroimaging, treatment regimens, and visual outcomes were analyzed. [Results] The cohort had a mean age of 50.4 years (range 31–66), with male predominance (84.6%, 11/13). Varicella zoster virus (VZV) was the leading pathogen (76.9%, 10/13), followed by herpes simplex virus type 1 (HSV-1), Epstein–Barr virus (EBV), and pseudorabies virus (PRV). Eight patients (61.5%) developed optic neuritis (ON) secondary to VZV infection, and five patients (38.5%) suffered from acute retinal necrosis (ARN), which was caused by VZV (*n* = 2), HSV-1 (*n* = 2), and PRV (*n* = 1). Bilateral involvement occurred in 46.2% (6/13) of patients. ARN was associated with the most severe visual loss. At the disease nadir, 46.2% of patients (6/13) presented with no light perception (NLP). Notably, five of these six NLP cases were diagnosed with ARN. Etiological confirmation was achieved in only 38.5% (5/13) of cases. mNGS of cerebrospinal and vitreous fluid, alongside aqueous humor PCR, are pivotal for diagnosing HSV-1/EBV mixed infections and rare PRV infection. All patients received antiviral therapy, 11 of whom (84.6%) were treated with intravenous antiviral agents. Glucocorticoids were administered as combination therapy to all patients. However, only one of eight VZV–ON eyes showed genuine visual improvement. In VZV–ARN, the initially involved eyes stayed NLP at final follow-up, while the fellow eyes recovered vision. Still, all non-VZV ARN patients had persistent bilateral NLP during follow-up. [Conclusions] Herpesvirus-associated visual impairment is dominated by VZV, manifests as ON or ARN, and carries a high risk of severe permanent vision loss—particularly in ARN. The emergence of zoonotic PRV underscores the need for heightened clinical vigilance. Diagnostic delays and insufficient interdisciplinary collaboration contribute substantially to poor outcomes.

## 1. Introduction

Herpesviruses are a group of enveloped, double-stranded DNA viruses with similar biological characteristics and are classified as Herpesviridae [1]. At present, more than 100 species have been found, which can be divided into three subfamilies of α, β, and γ according to biological and genomic characteristics. Alpha-herpesviruses include herpes simplex virus type 1 and type 2 (HSV-1/2), varicella zoster virus (VZV), and pseudorabies virus (PRV). β-herpesvirus is represented by human cytomegalovirus (HCMV). γ-herpesvirus (such as EB virus [EBV]) mainly infects lymphoid cells and can cause lymphoproliferative diseases [2].

Clinically, patients with herpes virus infection often visit dermatology, ophthalmology, neurology, and other departments simultaneously or in succession due to diverse systemic symptoms. This multidisciplinary rotation not only delays the diagnosis and treatment of some patients, causing serious consequences (e.g., permanent visual loss, fatal encephalitis), but it also fragments clinicians’ holistic understanding of the disease spectrum. Consequently, specialists frequently lack a systematic understanding of herpesvirus infections beyond their primary field of expertise. With the wide application of immunosuppressive agents and the aging of the population, the incidence of herpes virus infection is on the rise. It is noteworthy that PRV, also known as porcine herpesvirus type 1, has been shown to cross the species barrier and infect humans in recent years, leading to an acute retinal necrosis (ARN) syndrome and fatal encephalitis [3]. This emerging zoonotic transmission has expanded our understanding of the pathogenic spectrum of herpesviruses and highlighted the need for enhanced clinical vigilance.

Technological advances in metagenomic next-generation sequencing (mNGS) and neuroimaging have substantially improved the diagnosis of herpesvirus-triggered acute retinal necrosis syndrome and central nervous system infections, and have enriched our understanding of the pathogenesis and clinical features of acute optic neuritis (ON). Based on the above background, this study retrospectively analyzed the clinical data of patients with herpes-virus-infection-related visual impairment admitted to the Department of Neurology, Beijing Tongren Hospital, Capital Medical University, aiming to summarize their clinical characteristics, auxiliary examination characteristics, and prognosis, to improve clinicians’ overall understanding, diagnosis, and treatment of this entity.

## 2. Materials and Methods

### 2.1. Study Design and Participants

This was a single-center retrospective cohort study performed at the Department of Neurology, Beijing Tongren Hospital, Capital Medical University. Thirteen consecutive patients (19 affected eyes) diagnosed with visual impairment secondary to herpesvirus infection admitted between 1 January 2016 and 1 January 2025 were retrospectively reviewed. This study was approved by the Ethics Committee of Beijing Tongren Hospital (TRECKY2020-053, 9 May 2020).

Inclusion criteria:

1. Definite diagnosis of herpesvirus infection, confirmed by at least one of the following: (a) positive etiological test results (e.g., polymerase chain reaction [PCR], mNGS, or serological assays); (b) typical clinical manifestations (e.g., cutaneous herpes, encephalitic symptoms); or (c) characteristic epidemiological history.
2. Visual impairment as the primary clinical manifestation, with a clear temporal association between the onset of visual disturbance and herpesvirus infection (i.e., visual impairment occurred during the acute phase of herpesvirus infection or within a reasonable post-infection time window).
3. Typical optic neuritis presents with subacute monocular visual loss associated with pain during eye movement. Visual loss usually develops over hours or days [4].
4. Acute retinal necrosis diagnosed per the 2015 Japanese ARN Study Group criteria [5].

Exclusion criteria:

1. Confirmed diagnosis of neuromyelitis optica spectrum disorder according to 2015 international consensus diagnostic criteria [6].
2. Confirmed diagnosis of multiple sclerosis according to the 2010 or 2017 McDonald Criteria [7,8].
3. Presence of other infectious optic neuropathies (e.g., caused by bacteria, fungi, or non-herpesviruses) or non-infectious optic neuropathies (e.g., ischemic, toxic, metabolic, or autoimmune optic neuropathy).
4. History of primary ocular diseases that could independently induce visual impairment (e.g., glaucoma, age-related macular degeneration, retinal detachment, or diabetic retinopathy).
5. Incomplete clinical data precluding comprehensive analysis.

### 2.2. Study Methods

All enrolled patients underwent comprehensive neuro-ophthalmological and neurological examinations. Relevant clinical data were retrospectively retrieved from the hospital’s electronic medical record system, with the following information collected:

1. Demographic characteristics: gender and age at admission.
2. Clinical features: subtype of herpesvirus infection, time interval between rash onset and visual impairment, patterns of visual impairment (unilateral/bilateral, acute/subacute onset), and accompanying symptoms (e.g., headache, fever, cranial nerve palsy, or meningeal signs).
3. Auxiliary examinations: visual field assessment, fundus photography, brain and optic nerve magnetic resonance imaging (MRI) (including T1-weighted imaging [T1WI], T2-weighted imaging [T2WI], short tau inversion recovery [STIR], diffusion-weighted imaging [DWI], and gadolinium-enhanced sequences), routine cerebrospinal fluid (CSF) analysis (white blood cell count, glucose, and protein levels), and etiological tests (PCR, mNGS, or serological assays);
4. Treatment regimens: types of antiviral agents (e.g., acyclovir, ganciclovir), administration routes (intravenous or oral), time from visual impairment onset to initiation of antiviral therapy, and details of glucocorticoid use (dosage, route, and duration).

### 2.3. Visual Assessment

Best-corrected visual acuity (BCVA) at the nadir of visual impairment and last follow-up was measured using a Snellen decimal visual acuity chart at 6 m. For statistical analysis, Snellen decimal VA was converted to logMAR using the formula logMAR = −log_10_ (decimal VA) (logMAR 0 = 20/20; greater values indicate inferior visual acuity; 3.0 = no light perception). Visual impairment severity was categorized as mild (decimal VA > 0.5, logMAR < 0.30), moderate (decimal VA 0.1–0.5, logMAR 0.30–1.00), severe (decimal VA < 0.1, logMAR > 1.00), and NLP (logMAR 3.0). ΔlogMAR ≥ 0.2 indicates true visual improvement. 

### 2.4. Statistical Analyses

Descriptive statistics were used to summarize the study data. Continuous variables with non-normal distribution were expressed as median [M (range)], while categorical variables were presented as counts (percentages) [*n* (%)].

## 3. Results

A total of 13 patients with herpesvirus-infection-related visual impairment were enrolled in this retrospective cohort study, and their demographic characteristics are summarized in Table 1. The median age of the study population was 50 years old (range: 31–66 years). In terms of age stratification, five patients (38.5%) were younger than 50 years old; another five patients (38.5%) were aged 50–60 years; and the remaining three patients (23.0%) were over 60 years of age. The study population was predominantly male, accounting for 84.6% (11/13), while female patients accounted for 15.4% (2/13). VZV was the most prevalent pathogen, detected in 10 patients (76.9%). HSV-1 and EBV were identified in two patients (15.4%) and one patient (7.7%), respectively, with one patient presenting concurrent detection of HSV-1 and EBV. PRV was found in one case (7.7%). Regarding clinical phenotypes, eight patients (61.5%) developed ON secondary to VZV infection, and five patients (38.5%) suffered from ARN, which was caused by VZV (*n* = 2), HSV-1 (*n* = 2), and PRV (*n* = 1). Visual impairment occurred unilaterally in seven patients (53.8%) and bilaterally in six patients (46.2%). Among patients with unilateral lesions, all seven cases manifested ON; among the six bilateral cases, one presented with ON and five developed ARN.

### 3.1. Clinical Characteristics

All eight patients with optic neuritis (ON) had VZV-induced disease. Most exhibited a characteristic prodrome of facial or forehead zoster rash preceding unilateral visual deterioration. The entire ON subgroup was immunocompetent, except for one individual with pre-existing Takayasu arteritis who was maintained on immunosuppressive regimens comprising oral corticosteroids. Two ON patients carried a diagnosis of diabetes mellitus, and none of the eight subjects had undergone VZV vaccination. Concomitant cranial nerve palsies were observed in five ON patients (62.5%), all involving the oculomotor nerve (CN III); among these, two patients (25%) had additional involvement of the abducens nerve (CN VI) and trigeminal nerve (CN V).

In contrast to ON presentations, patients diagnosed with ARN commonly developed bilateral visual loss accompanied by constitutional symptoms such as fever and headache—a phenotype far more typical of ARN attributable to pathogens other than VZV. The severity of visual impairment was profound in our cohort: At the disease nadir, six out of thirteen patients (nine eyes, 46.2%) presented with no light perception (NLP). Notably, five of these six NLP cases were diagnosed with ARN, and the one remaining case was classified as ON. For the nine VZV cases (eight ON, one ARN), the median time from herpetic rash onset to visual impairment was 10 days (range, 3–60 days) (Table 2).

### 3.2. Etiological Testing

Positive etiological test results were obtained in five cases (38.5%), while negative or unavailable results were documented in eight cases (61.5%). The positive etiological findings included two cases of VZV, one case of HSV-1, one case of PRV, and one case of HSV-1/EBV coinfection (Table 2).

Among the 10 VZV-infected patients, only 2 (20.0%) were laboratory-confirmed by etiological testing, while the remaining 8 (80.0%) were clinically diagnosed according to typical herpetic cutaneous manifestations and a well-defined temporal relationship with the development of visual impairment. In Case 11, HSV was detected in CSF via mNGS, and aqueous humor PCR confirmed the viral subtype, yielding consistent results that validated the clinical diagnosis. Case 12 presented with viral encephalitis complicated by ARN: CSF mNGS was positive for EBV, and aqueous humor PCR further confirmed the coexistence of EBV and HSV.

### 3.3. Other Auxiliary Examinations

#### 3.3.1. Neuroimaging Examinations

**Figure 1:**
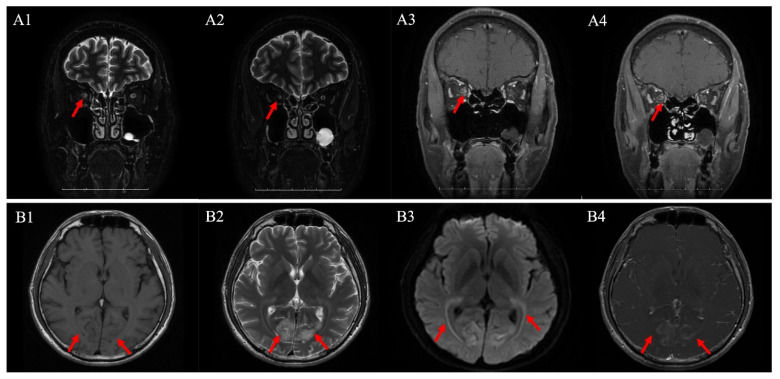
Orbital and cranial MRI findings of Case 11. A, Orbital MRI: Coronal short tau inversion recovery (STIR) sequences (**A1**,**A2**) and gadolinium-enhanced T1-weighted imaging (T1WI) (**A3**,**A4**) demonstrate hyperintensity in the intraorbital, intracanalicular, and intracranial segments of the right optic nerve (red arrows); the left optic nerve shows normal morphology and signal intensity. B, Cranial MRI: Patchy lesions are visualized in the bilateral occipital cortex and subcortical white matter, manifesting as hypointensity on T1WI ((**B1**), red arrow), hyperintensity on T2WI ((**B2**), red arrow), restricted diffusion on diffusion-weighted imaging (DWI) ((**B3**), red arrow), and significant enhancement on gadolinium-enhanced T1WI ((**B4**), red arrow).

Most patients with VZV–ON showed unilateral visual impairment, and orbital MRI frequently revealed inflammatory lesions of the optic nerve, optic nerve sheath, or cavernous sinus. Among these, one case showed concurrent meningeal enhancement (Case 9), and one case had pons and medulla involvement (Case 4). VZV–ARN mainly affects the optic nerve and its sheath on imaging. In contrast, non-VZV–ARN exhibited MRI features consistent with viral encephalitis, including cerebral parenchymal lesions. Notably, Case 11 showed extensive involvement of the visual pathway, with lesions extending from the retina, optic nerve, optic chiasm, and optic radiation to the visual cortex (Figure 1).

#### 3.3.2. Fundus Examination

**Figure 2:**
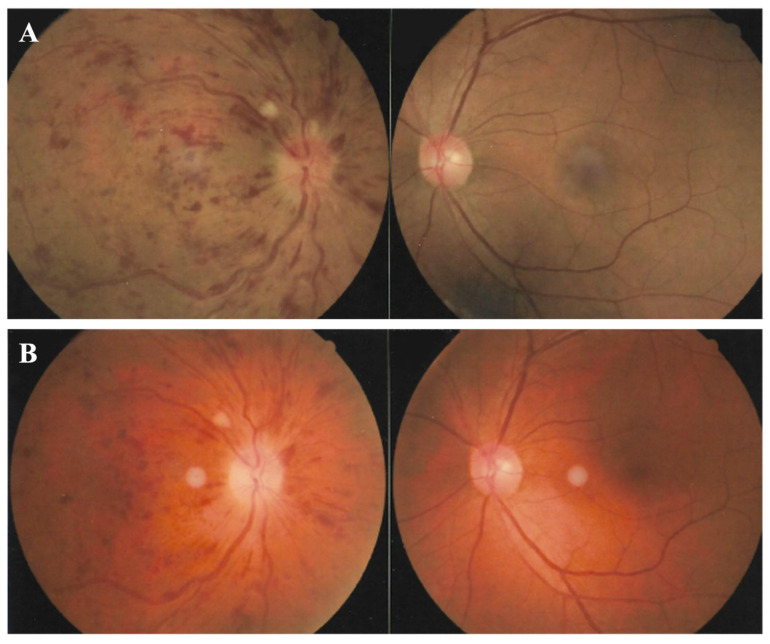
Fundus photographs of Case 8. (**A**) Initial presentation: The right eye shows optic disk edema, blurred margins, and punctate/linear hemorrhages; the left eye (OS) demonstrates a normal fundus. (**B**) Two weeks after antiviral therapy, optic disk edema in the right eye is alleviated, with partial absorption of hemorrhages.

Most patients with optic neuritis showed unremarkable fundus findings. In this cohort, eight of nine eyes (88.9%) affected by ON exhibited clear optic disk margins and normal coloration, while only one eye (11.1%) presented with blurred disk margins and mild pallor. In contrast, patients with acute retinal necrosis displayed optic disk edema, blurred margins, and peripapillary hemorrhage. Case 8 was diagnosed with ARN in the right eye, characterized by optic disk edema, ill-defined margins, and punctate/linear hemorrhages; the left eye was diagnosed with ON with normal fundus appearance (Figure 2).

#### 3.3.3. Lumbar Puncture and Cerebrospinal Fluid (CSF) Analysis

Lumbar puncture was performed in all 13 patients. The intracranial pressure (ICP) ranged from 80 to 235 mmH_2_O, with a median of 127.5 mmH_2_O. Elevated ICP (>180 mmH_2_O) was recorded in 3 of 12 patients with available data (25.0%), while 9 patients (75.0%) had normal ICP (80–180 mmH_2_O); ICP data were unavailable for 1 patient transferred from an external institution. The CSF white blood cell count ranged from 0 to 162 × 10^6^/L (median, 22 × 10^6^/L; normal range, 0–8 × 10^6^/L). CSF protein levels varied from 12.1 to 86.4 mg/dL (median, 63.6 mg/dL; normal range, 15–45 mg/dL). (Table 2).

### 3.4. Treatment and Prognosis

The treatment regimens and prognostic outcomes of the 13 patients are summarized in Table 2. All patients received combined therapy with glucocorticoids and antiviral agents. Regarding antiviral administration routes, 11 patients (84.6%) received intravenous antiviral therapy, and 2 patients (15.4%) were treated with oral antiviral agents alone. Patients with PRV-AVN were given intravenous acyclovir in the encephalitic phase before visual impairment developed. For the remaining 10 patients, the time lag between the onset of visual loss and the administration of intravenous acyclovir was 5–39 days (median, 16 days). Only one patient (10) initiated treatment within 1 week of visual loss. Glucocorticoids were administered to all 13 patients: 5 patients (38.5%) received high-dose pulse therapy (methylprednisolone equivalent ≥500 mg/day); 3 patients (23.0%) received low-dose intravenous glucocorticoids (≤80 mg/day); and 5 patients (38.5%) received oral prednisone (1 mg/kg/day). Among the eight eyes affected by VZV–ON, only one achieved genuine visual improvement. In patients with VZV–ARN, the initially affected eye remained NLP at the last follow-up, whereas visual acuity improved in the contralateral eye. By contrast, all patients with non-VZV ARN maintained NLP bilaterally throughout follow-up.

## 4. Reference Cases

### 4.1. Case 7

A 50-year-old male was admitted to our hospital with chief complaints of left facial herpes for 14 days and bilateral visual acuity decline for 8 days. His past medical history included hypertension and chronic rhinitis. On admission, the BCVA was 20/70 in the right eye and NLP in the left eye. Both pupils were equal in size and round, with a diameter of 3 mm; the direct and consensual light reflexes of the left eye were sluggish. Fundus examination of both eyes showed no obvious abnormalities, with clear optic disk margins and normal coloration.

**Figure 3:**
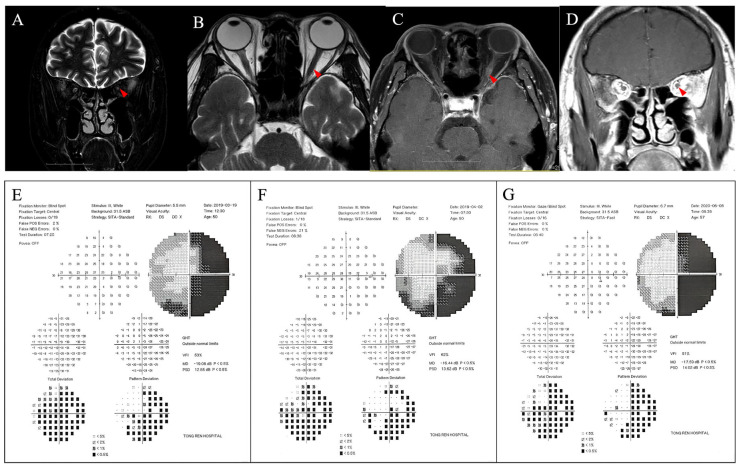
Neuroimaging and visual field changes in Case 7. (**A**–**D**) Orbital MRI: (**A**) Coronal short tau inversion recovery (STIR) sequence shows atrophy and increased signal intensity of the intraorbital segment of the left optic nerve (red arrowhead). (**B**) Axial T2WI shows atrophy of the intraorbital segment of the left optic nerve (red arrowhead). (**C**) Gadolinium-enhanced axial T1WI demonstrates patchy enhancement of the left optic nerve sheath (red arrowhead) and no enhancement of the right optic nerve. (**D**) Gadolinium-enhanced coronal T1WI demonstrates atrophy but without prominent enhancement of the left optic nerve (red arrowhead). (**E**) Visual field at admission (Day 16): right homonymous temporal hemianopsia. (**F**) Visual field after 2 weeks of treatment (Day 30): reduced range of temporal defect in the right eye. (**G**) Visual field at 3-month follow-up: further improvement of the right visual field.

Orbital MRI revealed atrophy of the intraorbital segment of the left optic nerve with abnormal enhancement of the optic nerve sheath; the intraorbital segment of the right optic nerve showed increased signal intensity on T2WI without abnormal enhancement (Figure 3A–D). Visual field testing demonstrated right homonymous temporal hemianopsia (Figure 3E), while the left eye could not complete the examination due to NLP. Neither pattern visual evoked potential (P-VEP) nor flash visual evoked potential (F-VEP) waveforms were recordable from the left eye. P-VEP amplitudes of the right eye were within normal limits. The P100 latencies of P-VEP recorded at O1, Oz, and O2 were prolonged in his right eye, measuring 120 ms, 133 ms, and 134 ms, respectively (normal range 90–120 ms). The P100 latencies of F-VEP recorded at O1, Oz, and O2 in his right eye, measuring 153 ms, 150 ms, and 149 ms, respectively (normal range 100–150 ms). Comprehensive serum testing for glial-specific autoantibodies associated with ON, including aquaporin-4 (AQP4) IgG, myelin oligodendrocyte glycoprotein (MOG) IgG, glial fibrillary acidic protein (GFAP) IgG, and flotillin IgG, yielded negative results. Lumbar puncture showed a normal intracranial pressure of 125 mmH_2_O, an elevated CSF white blood cell count of 40 × 10^6^/L (normal range 0–8 × 10^6^/L), an increased CSF protein concentration of 86.4 mg/dL (normal range 15–45 mg/dL), and an elevated 24 h intrathecal synthesis rate of 3.50 mg/d (normal range −30–0.7 mg/d). CSF mNGS detected 2 specific sequences of VZV.

The patient received oral methylprednisolone (1 mg/kg/day) combined with antiviral therapy, including intravitreal injection of ganciclovir, topical acyclovir eye drops, and neurotrophic support. During hospitalization, visual acuity did not improve, but visual field testing showed a reduced temporal defect range in the right eye (Figure 3F,G). Before discharge, re-examination of CSF revealed a white blood cell count of 52 × 10^6^/L (normal range 0–8 × 10^6^/L), a protein concentration of 67.6 mg/dL (normal range 15–45 mg/dL), a 24 h intrathecal synthesis rate of −1.05 mg/d (normal range −30–0.7 mg/d), and no VZV-specific sequences detected by mNGS.

This case presented a typical course of bilateral optic neuritis following VZV infection, with unilateral facial herpes zoster preceding sequential involvement of both eyes. VZV spreads to the contralateral optic nerve via the optic chiasm, leading to bilateral optic neuritis and characteristic temporal visual field defects. Neuroimaging showing optic chiasm involvement supports the mechanism of direct viral invasion along the visual pathway.

### 4.2. Case 13

A 49-year-old male, an individual pork vendor, was admitted to our hospital with chief complaints of fever accompanied by loss of consciousness and convulsions 4 months ago and progressive bilateral visual acuity decline for 1.5 months. He had a past medical history of hypertension.

**Figure 4:**
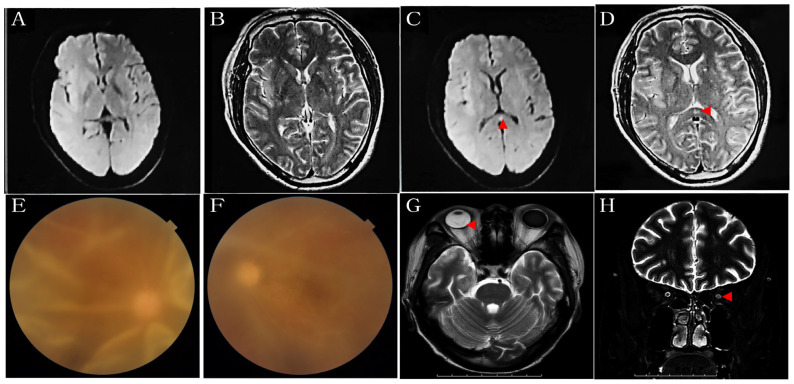
MRI findings of Case 13. Brain MRI of Case 13 during the acute stage of encephalitis showed DWI and T2 hyperintense lesions in the right temporal lobe (**A**,**B**) and splenial corpus callosum (arrowheaded) (**C**,**D**). Fundus photography of both eyes showed retinal necrosis and detachment (**E**,**F**). MRI showed “V “shaped detachment of his right retina (arrowheaded) (**G**) and signal increased in the left optic nerve (arrowheaded) (**H**).

Four months prior to admission, he presented with fever (peak temperature 40.0 °C) without identifiable predisposing factors, accompanied by headache and nausea. Within a few days, he experienced recurrent loss of consciousness with limb convulsions. Brain MRI in the acute phase showed DWI- and T2-hyperintense lesions in the right temporal lobes (Figure 4A,B) and splenial corpus callosum (Figure 4C,D). Lumbar puncture revealed a CSF pressure of 180 mmH_2_O, a white blood cell count of 39 × 10^6^/L (normal range 0–8 × 10^6^/L), a protein concentration of 45 mg/dL (normal range 15–45 mg/dL), and normal glucose. Serum and CSF autoimmune encephalitis antibody panels were negative. He was diagnosed with “viral encephalitis” and treated with intravenous acyclovir (10 mg/kg, q8h), methylprednisolone pulse therapy (500 mg/day for 3 days, followed by sequential oral administration), intravenous immunoglobulin (0.4 g/kg/day for 5 days), as well as sedation, antiepileptic therapy, and tracheotomy with mechanical ventilation. His condition stabilized after 1.5 months of treatment, and he was discharged with residual recent memory impairment. Pre-discharge re-examination of CSF showed a white blood cell count of 11 × 10^6^/L (normal range 0–8 × 10^6^/L) and a protein concentration of 80 mg/dL (normal range 15–45 mg/dL). Two months after discharge (i.e., 3.5 months after the onset of encephalitis), he developed progressive bilateral visual acuity decline, which rapidly progressed to NLP in both eyes within 1.5 months. Fundus photography of both eyes showed retinal necrosis and detachment (Figure 4E,F). MRI showed “V” shaped detachment of the right retina (Figure 4G) and signal increased in the left optic nerve (Figure 4H). There were no prodromal symptoms such as fever or headache at the onset of visual impairment.

A neurology consultation was requested, and a lumbar puncture was repeated. The intracranial pressure was 140 mmH_2_O, and the CSF was colorless and transparent. The white blood cell count was 2 × 10^6^/L (normal range 0–8 × 10^6^/L), the protein concentration was 82 mg/dL (normal range 15–45 mg/dL), the glucose level was 3.2 mmol/L, and the chloride level was normal. CSF etiological mNGS was negative for DNA viruses. Autoimmune encephalitis antibody panel and autoimmune glial cell antibodies (AQP4, MOG, GFAP) were all negative. Vitreous fluid mNGS detected pseudorabies virus (PRV, suid herpesvirus 1) as the only virus, with 153 detected sequences and a genomic coverage of 4.33%. Due to limited conditions, PRV antibody testing in CSF and serum was not performed. Combined with the epidemiological history (occupation as a pork vendor) and clinical course, the patient was diagnosed with ARN caused by PRV infection.

In this case of PRV encephalitis, cerebrospinal fluid mNGS was negative during the recovery period, whereas vitreous PRV was positive, with visual impairment, suggesting that the virus may have anterogradely disseminated to the retina along the optic nerve and reactivated when the patient’s immune status changed.

## 5. Discussion

Herpesviruses are characterized by large double-stranded DNA genomes, complex enveloped structures, and the ability to establish latent infections throughout the host’s lifespan. Mammalian herpesviruses are classified into three subfamilies (α, β, and γ) based on genomic composition, the cell types colonized during latent infection, and the duration of the productive replication cycle. Among these, α-herpesviruses exhibit the broadest host range and display neurotropism. Once invading the peripheral or central nervous system (CNS), α-herpesviruses can spread along chains of synaptically connected neurons [1].

In the present retrospective study, VZV infection was the most prevalent among all 13 patients, accounting for 10 cases. Herpes zoster (HZ) is caused by the reactivation of VZV that has been latently infected in sensory ganglia (cranial nerves or dorsal root ganglia) following primary varicella infection in childhood [9]. The incidence of HZ increases with age, which is associated with the decline in VZV-specific cellular immunity [10]. A meta-regression analysis of data from 59 studies across 29 countries estimated that the global incidence of HZ is 5.15 per 1000 individuals in the 50–54 age group and 11.27 per 1000 individuals in those aged ≥85 years [11].

In this study, eight patients developed optic nerve involvement on the basis of HZO, and some patients had concurrent oculomotor nerve and/or abducens nerve involvement. HZO specifically refers to HZ involving the ophthalmic division of the fifth cranial nerve (trigeminal nerve) following VZV reactivation. Among the three major branches of the ophthalmic division, the frontal branch is most affected, but the nasociliary and lacrimal branches may also be involved [12]. In addition to innervating the ocular surface, iris, and choroid, the nasociliary branch innervates the skin surfaces of the eyelid and nose. Involvement of this branch presents as Hutchinson’s sign, which refers to herpetic lesions along the nose and strongly suggests intraocular involvement [13]. Approximately 4–20% of individuals with HZ develop HZO [14]. Studies have reported that approximately 70% of HZO cases occur in individuals > 50 years of age [15], while the proportion in this cohort was 55.6%. In rare instances, zoster sine herpete is characterized by ocular involvement without skin lesions, as demonstrated in Case 10 of our study.

VZV rarely induces optic neuropathy, which develops in <0.5% of patients with HZO [16]. The pathophysiology of VZV-induced optic neuropathy is not fully understood and may include direct optic nerve infection via the cavernous sinus or hematogenous route, as well as indirect causes such as inflammatory demyelination, optic perineuritis, vasculitis causing ocular ischemia, and immune responses secondary to extra-orbital infections leading to optic disk edema [15,17,18]. VZV-induced optic neuropathy may occur before the onset of HZO (preceding the rash in some cases), during HZO, or as an acute late complication up to 10 weeks after HZO onset. Given this, aggressive treatment during the initial viral reactivation may reduce the incidence of subsequent optic neuropathy [19].

Patients with VZV optic neuropathy may present with acute visual loss with or without eye movement pain (which may be unilateral or bilateral). Other potential clinical findings include a relative afferent pupillary defect (RAPD), as well as visual field defects and dyschromatopsia. Optic disk edema on the contralateral side to the affected HZO side, or even bilateral involvement, has also been reported [20,21].

Regarding indirect causes, some scholars have hypothesized that VZV plays a triggering role in the multifactorial development of neuromyelitis optica spectrum disorder, multiple sclerosis, and myelin oligodendrocyte glycoprotein disease [22,23,24]. One mechanism may be that VZV promotes blood–brain barrier disruption (as indicated by CSF serum albumin ratio in HZ), thereby exposing CNS antigens to autoimmune responses [22]. Other etiologies of optic neuritis, such as MOG-IgG- or AQP4-IgG-related disorders, should be considered as important differential diagnoses.

For optic neuropathy, CSF PCR may be considered to confirm active VZV reactivation. Notably, CSF etiological PCR testing is often negative in the early stage of the disease. However, with disease progression, repeated testing often yields positive results despite empirical acyclovir treatment. When complicated by encephalitis and meningitis, CSF analysis usually shows pleocytosis (>5 × 10^6^ cells/L), predominantly lymphocytes. CSF glucose is usually normal, while protein may be normal or moderately elevated. In cases complicated by uveitis, aqueous humor paracentesis for etiological examination may also be considered [25].

ARN is a rare and severe ophthalmic disease characterized by panuveitis, retinal necrosis, and a high rate of retinal detachment [26]. It is widely accepted that the main cause of ARN is VZV, followed by HSV-1 and HSV-2, while CMV is a less common etiological agent [27]. Although EBV has been reported to be associated with ARN in previous studies [28], it is considered non-pathogenic in most cases [29]. Common manifestations of ARN include anterior uveitis, scleritis, vitritis, necrotizing retinitis, occlusive vasculitis, and optic disk edema [30]. Factors associated with visual prognosis include VZV infection [31], retinitis [32], delayed diagnosis [33], poor visual acuity at presentation [34], and optic nerve involvement [32]. For diagnosis, PCR testing of intraocular fluid for viruses has high sensitivity (79–100%), and aqueous humor is preferred due to its safe accessibility; quantitative PCR can also be used to monitor treatment response and viral load [26,27,35,36].

The most common ocular complication of HSV infection is ARN [37],. HSV accounts for 20–30% of ARN cases [33,34,38,39], mostly occurring in adults aged 20–40 years or children [40], and is often associated with viral encephalitis (especially herpes simplex encephalitis [HSE]) [41]. In contrast, VZV–ARN mainly occurs in individuals over 50 years of age [38]. Approximately 24% of cases have a history of HSE, and 31.3% of HSV–ARN occurring after HSE involve both eyes [42], with an interval between encephalitis and ARN onset usually of 1–5 months [43]. Patients with HSV–ARN (regardless of previous CNS infection history) often present with posterior pole involvement, such as optic disk edema, arterial sheathing, and retinal hemorrhages, which are characteristic vascular inflammatory changes that may occur before the appearance of peripheral retinal necrosis or serve as the main manifestations (e.g., early macular involvement, vitreous hemorrhage) [44]. Clinically, when arterial sheathing is accompanied by optic disk edema, a viral etiology should be highly suspected, and aqueous humor PCR testing may be performed in cases of acute onset or deterioration. Cases of optic neuritis caused by HSV infection are less common than those caused by ARN. There were no cases of optic neuritis caused by HSV infection in this report. Previous studies have suggested that the pathogenesis of unilateral optic neuritis following HSV infection is driven by a virus-triggered immune response (rather than direct viral infection or CNS demyelinating disease) and is responsive to glucocorticoid treatment [45,46,47]. The HSV-1/EBV coinfection in Case 12 exemplifies dual viral compartmentalization across anatomically distinct sites. CSF mNGS detected EBV, while aqueous humor PCR confirmed the concurrent presence of HSV-1 and EBV. EBV, a γ-herpesvirus establishing latency in B lymphocytes, typically causes infectious mononucleosis-related encephalitis or primary CNS lymphoma, and is considered non-pathogenic in most ARN cases. Therefore, this case is best classified as HSV-1-associated ARN with concurrent EBV presence and possible EBV-associated encephalitis. It also underscores a critical diagnostic pitfall. Single-compartment testing would have yielded misleadingly incomplete results—CSF-only analysis would have missed HSV-1, compromising antiviral coverage. We recommend parallel CSF mNGS and intraocular fluid PCR for patients with ARN accompanied by encephalitic features or atypical bilateral posterior-predominant retinitis.

PRV is an α-herpesvirus that can infect a variety of wild and domestic animals and can cause neurological diseases in humans. Notably, occupational groups related to the pig industry are highly susceptible. The PRV case in this report was a pork vendor, which is consistent with the corresponding epidemiological history. The virus is neurotropic and often causes severe CNS complications such as encephalitis after infecting humans. In recent years, multiple cases of human encephalitis caused by PRV have been reported [48,49,50,51,52]. These patients are often accompanied by severe visual impairment, including ocular complications such as necrotizing retinitis. Very few patients present only with fever and ocular symptoms without evidence of intracranial infection or encephalitis manifestations [48]. The retinal detachment rate of PRV–ARN is higher than that of HSV–ARN (37%) and VZV–ARN (46%) [34], suggesting a worse visual prognosis for PRV–ARN. In previous reports, only a few patients showed improved visual acuity after treatment (e.g., from counting fingers to 0.2), but the proportion of patients recovering useful visual acuity is very low [53,54,55].

Systemic antiviral therapy is the core intervention for ARN, which can improve retinitis inflammation and reduce contralateral eye involvement [56,57]. Initial treatment may include intravenous acyclovir or oral prodrugs valacyclovir and famciclovir, which are increasingly used due to their convenience and comparable efficacy [35,58,59]. For acyclovir-resistant cases, foscarnet, which does not require viral kinase activation, can be used, but renal toxicity should be monitored [60]. Ganciclovir is also effective, but attention should be paid to its myelosuppressive risk [59,60]. To enhance local control, intravitreal antiviral therapy (such as ganciclovir or foscarnet) is usually combined, which can immediately reduce intraocular viral load and improve prognosis but cannot replace systemic therapy to prevent contralateral eye onset [34,61].

On the premise of effective antiviral therapy (usually 24–48 h after the initiation of antiviral treatment), systemic glucocorticoids (such as oral prednisone) can be combined to control severe inflammatory responses, but attention should be paid to their potential to promote viral replication [59,62,63]. Regarding long-term antiviral prophylaxis, there is currently no unified standard. Long-term oral valacyclovir or acyclovir may be considered to prevent contralateral eye involvement and recurrence, but extended treatment beyond one year may not bring additional benefits [57,64]. The dosage needs to be individualized according to the patient’s visual status, renal function, and comorbidities (e.g., valacyclovir 500 mg twice daily) [65]. Previous reports have shown that cases of optic neuritis caused by herpesvirus infection respond poorly to steroids but achieve significant efficacy through intravenous immunoglobulin (IVIG) treatment, suggesting that immune-mediated mechanisms also play an important role in the pathogenesis [37,44]. IVIG immunotherapy may be considered for patients with poor response to antiviral therapy and glucocorticoid treatment or severe conditions.

Several limitations should be acknowledged. This single-center retrospective cohort study had a small sample size without a control group or inferential statistical analyses, which limited its external validity. Meanwhile, although eight patients with VZV infection were diagnosed clinically based on typical herpetic cutaneous lesions and a clear temporal correlation with the onset of ipsilateral visual impairment, a risk of etiological misclassification still existed. In addition, there was marked heterogeneity in the timing of intravenous acyclovir initiation across subjects. Furthermore, insufficient long-term follow-up data prevented us from evaluating sustained visual function and the risk of disease recurrence.

## 6. Conclusions

In summary, herpesvirus-infection-related visual impairment is a neurological complication caused by different subtypes of herpesviruses, including VZV, HSV, and PRV. It is characterized by diverse clinical manifestations, diagnostic challenges, delayed treatment, and overall poor prognosis. Some cases may present as delayed visual impairment occurring during the recovery phase of viral encephalitis. The diagnosis faces multiple challenges, and treatment delay is common, with a significant lag in the initiation of antiviral therapy. The overall prognosis is poor, with most patients having unsatisfactory visual recovery, especially those with acute retinal necrosis.

## Figures and Tables

**Table 1:** Demographic characteristics of patients with herpes-associated visual impairments.

	Number of Patients, N = 13	%
Age		
Median age	50	
Intervals		
<50	5	38.5
50 to 60	5	38.5
>60	3	23.0
Gender		
Male	11	84.6
Female	2	15.4
Virus involved		
VZV	10	76.9
HSV-1	2 *	15.4
EBV	1 *	7.7
PRV	1	7.7
Visual impairment		
ON	8(VZV)	61.5
ARN	5(VZV/HSV-1/PRV: 2/2/1)	38.5
Laterality		
Unilateral	7(ON)	53.8
Bilateral	6(ON/ARN:1/5)	46.2

Abbreviations: ARN = acute retinal necrosis; EBV = Epstein–Barr virus; HSV-1 = herpes simplex virus type 1; ON = optic neuritis; PRV = pseudorabies virus; VZV = varicella zoster virus. * There was one case of HSV-1 and EBV coinfection.

**Table 2:** The treatment regimens and prognostic outcomes of the 13 patients.

No.	Sex/Age	Presentation; Past Medical History; Immune Status	Vision Loss Reason	Virus	VA * (OD/OS)			Treatment #	mNGS	Other Etiological Tests	CSF			MRI Abnormalities
					Nadir	LFU	Time Interval				WBC * 10^6^/L (0–8)	PRO mg/dL (15–45)	ICP mmH_2_O	
1	M/31	left forehead shingles/30 d later vision loss; previously healthy; immunocompetent	ON	VZV	0/2	0/1.9	17 d	Day 13 started acyclovir 0.75 g IV q8 h × 14 d; IVIg; steroids 1 g IV qd × 3 d, 0.5 g IV qd × 3 d, then oral	ND	NR	1	29.8	200	left optic nerve
2	M/63	left forehead and facial shingles/6 d later vision loss/restricted eye movements/ptosis; HT; immunocompetent	ON	VZV	0/2	0/2	31 d	Day 39 started acyclovir 0.5 g IV q8 h × 21 d, then oral; ganciclovir ophthalmic gel; oral steroids	ND	NR	6	77.8	80	left optic nerve sheath/cavernous sinus
3	M/53	left forehead shingles/10 d later vision loss/restricted eye movements/ptosis; HT and DM; immunocompetent	ON	VZV	2/2	2/2	15 d	Day 10 started acyclovir 0.5 g IVq8 h × 10 d, then oral; steroids 80 mg IV qd × 10 d, then oral	ND	NR	1	46.5	185	left cavernous sinus/orbital apex
4	F/66	right forehead shingles/10 d later vision loss/restricted eye movements/ptosis; previously healthy; immunocompetent	ON	VZV	0.7/0	0.7/0	28 d	Day 35 started acyclovir 0.5 g IV q8 h × 21 d, then oral; ganciclovir ophthalmic gel; steroids 80 mg IV qd × 14 d, then 40 mg IV qd × 8 d, then oral	CSF (-)	NR	40	64.3	120	right optic nerve and sheath/cavernous sinus/pons/medulla
5	M/45	left forehead shingles/ 17 d later vision loss; DM; immunocompetent	ON	VZV	0/2	0/1.9	14 d	oral penciclovir; ganciclovir ophthalmic; steroids 1 g IV qd × 3 d, 0.5 g IV qd × 3 d, then oral	ND	NR	0	39.4	120	left optic nerve
6	M/37	left facial shingles/60 d later vision loss/ptosis/dilated pupil; previously healthy; immunocompetent	ON	VZV	0/2	0/0.5	21 d	oral penciclovir; oral steroids	ND	NR	70	12.1	180	left optic nerve/oculomotor nerve/maxillary nerve
7	M/50	left forehead and nose shingles/6 d later bilateral vision loss; HT and rhinitis; immunocompetent	ON	VZV	0.5/3	0.5/3	33 d	Day 5 started acyclovir 0.75 g IV q8 h × 21 d; ganciclovir intravitreal injection; oral steroids	CSF: VZV	CSF: HSV-1/2-IgG	40	80.4	125	bilateral optic nerves and left cavernous sinus
8	M/54	right facial shingles/ 3 d later bilateral vision loss; previously healthy; immunocompetent	ARN	VZV	3/0.7	3/0	17 d	Day 16 started acyclovir 0.75 g IV q8 h × 14 d; ganciclovir intravitreal injection; steroids 0.5 g IV × 1 d	CSF (-)	CSF: HSV-1/2-IgG; CMV-IgG	65	54.1	110	right optic nerve and sheath
9	F/43	left ptosis/shingles/ 4 d later vision loss; Takayasu arteritis; immunocompromised	ON	VZV	0/0	0/0	1440 d	Day 18 started acyclovir 0.5 g IV q8 h × 21 d; ganciclovir ophthalmic gel; oral steroids	CSF (-)	CSF (-)	22	63.6	150	left optic nerve sheath/cavernous sinus; meninges
10	M/59	left vision loss then right; HT and DM; immunocompetent	ARN	VZV	0.4/3	0/3	810 d	Day 15 started acyclovir 0.5 g IV q8 h × 8 d, then 1.0 g IV q8 h × 14 d, then oral penciclovir; ganciclovir ophthalmic gel; intravitreal ganciclovir injection; oral steroids	CSF: VZV	Serum: VZV- IgG; CSF: VZV-IgG; AH: VZV	162	54.6	235	right optic nerve and left optic nerve sheath
11	M/41	headache, fever, 10 d later bilateral vision loss; previously healthy; immunocompetent	ARN	HSV-1	3/3	3/3	34 d	Day 13 started acyclovir 0.5 g IV q8 h × 21 d, then oral; intravitreal ganciclovir, steroids 80 mg IV qd × 5 d, then 40 mg IV qd × 3 d, then oral	CSF: HSV-1	AH: HSV-1	90	65.4	120	right optic nerve; bilateral optic radiations/occipital cortices
12	M/64	headache, fever, 3 d later bilateral vision loss, cognitive decline, abnormal behavior; previously healthy; immunocompetent	ARN	HSV-1/EBV	3/3	3/3	20 d	Day 34 started acyclovir 0.5 g IV q8 h × 7 d, then oral; steroids 1 g IV qd × 3 d, then 0.5 g IV qd × 3 d, then oral tapered	CSF: EBV	AH: HSV-1/EBV	10	86.4	130	bilateral optic nerve/optic chiasm/optic tracts/thalamus; right optic radiation/occipital lobe; left insula/temporal lobe
13	M/49	fever, convulsions, cognitive decline, 75 d later bilateral vision loss; HT; immunocompetent	ARN	PRV	3/3	3/3	45 d	Before vision loss acyclovir 0.5 g IV q8 h × 21 d; steroids 0.5 g IV qd × 3 d, then oral tapered; IVIg	CSF (-) VF: PRV	CSF (-)	2	82	NA	bilateral optic nerves, right temporal lobe and splenium of the corpus callosum

Abbreviations: AH = aqueous humor; CSF = cerebrospinal fluid; DM = diabetes mellitus; EBV = Epstein–Barr virus; F = female; HSV-1 = herpes simplex virus type 1; HT = hypertension; ICP = intracranial pressure; IV = intravenous; IVIg = intravenous immunoglobulin; LFU = last follow-up; M = male; NA = not available; mNGS = metagenomic next-generation sequencing; MRI = magnetic resonance imaging; ND = not done; NR = not recorded; No. = number; OD = oculus dexter; ON = optic neuritis; OS = oculus sinister; PRO = protein; PRV = pseudorabies virus; VA = visual acuity; VF = vitreous fluid.; VZV = varicella zoster virus; WBC = white blood cell. The ‘NR’ in ‘Other etiological testing’ refers to tests for identified disease-causing pathogens, such as VZV and HSV, which were not recorded, rather than a complete lack of pathogenic testing. * X/Y = logMAR visual acuity of the right eye (OD, X) and left eye (OS, Y). Lower values indicate better visual acuity. # The “Day” entries recorded in the Treatment section document the number of days following visual impairment onset when intravenous antiviral therapy was initiated.

## Data Availability

The data presented in this study are available on request from the corresponding author due to privacy restrictions and hospital regulations protecting patient confidentiality.
